# A New Sixth Order Method for Nonlinear Equations in *R*

**DOI:** 10.1155/2014/890138

**Published:** 2014-01-23

**Authors:** Sukhjit Singh, D. K. Gupta

**Affiliations:** Department of Mathematics, Indian Institute of Technology Kharagpur, Kharagpur 721302, India

## Abstract

A new iterative method is described for finding the real roots of nonlinear equations in *R*. Starting with a suitably chosen *x*_0_, the method generates a sequence of iterates converging to the root. The convergence analysis is provided to establish its sixth order of convergence. The number of iterations and the total number of function evaluations used to get a simple root are taken as performance measure of our method. The efficacy of the method is tested on a number of numerical examples and the results obtained are summarized in tables. It is observed that our method is superior to Newton's method and other sixth order methods considered.

## 1. Introduction

One of the most important and challenging problems in science and engineering is to find real roots of nonlinear equations in *R*. There exist a large number of applications that give rise to thousands of such equations depending on one or more parameters. For example, the kinetic theory of gases, elasticity, and other applied areas are reduced to solving these equations. With the development of computer S/W and H/W, this problem has gained added advantages. This paper is concerned with the iterative methods and their convergence analysis for finding a simple real root *α*; that is, *f*(*α*) = 0 and *f*′(*α*) ≠ 0 of

(1) $$\begin{array}{c} f\left(x\right)=0, \end{array}$$

where *f* : *D* ⊂ *R* → *R* is the continuously differentiable real function on an open interval *D*. This problem is extensively studied by many researchers [1–5] and many methods along with their convergence analysis are derived. For good reviews of these methods, one can refer to excellent books by Ortega and Rheinboldt [6], Ostrowski [2], Traub [7], and many others. The well-known Newton's method used to find *α* starts with an initial approximation *x*_0_ near to the root *α* and generates a sequence of iterates {*x*_*k*_} converging quadratically to the root. It is given for *k* = 0,1, 2,…, by

(2) $$\begin{array}{c} x_{k+1}=x_{k}-\frac{f\left(x_{k}\right)}{f^{\prime }\left(x_{k}\right)}. \end{array}$$

If the efficiency index [8] of an iterative method is defined as *p* ^1/*m*^, where *p* is the order of the method and *m* is the number of functions evaluations per iteration, then the efficiency index of Newton's method is 1.414. A number of ways are considered by many researchers to improve the local order of convergence of Newton's method at the expense of additional evaluations of functions, derivatives, and changes in the points of iterations. All these modifications are in the direction of increasing the local order of convergence with the view of increasing their efficiency indices. For example, Frontini and Sormani [9] developed new modifications of Newton's method to produce iterative methods with order of convergence of three and efficiency index of 1.442. With the same efficiency index, Traub [7] developed a third order method requiring evaluations of one function and two first derivatives per iteration. In [3], a family of third order methods is given which requires evaluations of one function, one first derivative, and one second derivative per iteration. Starting with a suitably chosen initial approximation to the root *α*, it is given for *k* = 0,1, 2,… by

(3) $$\begin{array}{c} x_{k+1}=x_{k}-\left(1+\frac{1}{2}\frac{L_{f}\left(x_{k}\right)}{1-\beta L_{f}\left(x_{k}\right)}\right)\frac{f\left(x_{k}\right)}{f^{\prime }\left(x_{k}\right)}, \end{array}$$

where *L*_*f*_(*x*_*k*_) = (*f*′′(*x*_*k*_)*f*(*x*_*k*_))/(*f*′(*x*_*k*_))^2^ and *β* is a parameter. One can easily see that for particular values of *β* = 0,1/2, and 1, Chebyshev's, Halley's, and Super-Halley's methods are special cases of this family. These methods require evaluation of *f*, *f*′, and *f*′′ per iteration and hence their efficiency indices are also 1.442. But the disadvantage of these methods is that they involve the evaluation second order derivative which is either computationally difficult to compute or remains unbounded. King [1] developed a family of fourth order methods for solving (1) requiring evaluation of one function and its derivative at the starting point of each step and of the function alone at the so-called Newton point. Ostrowski [2] developed both third and fourth order methods each requiring evaluations of two functions and one derivative per iteration leading to its efficiency index equal to 1.587.

Recent trend is to develop sixth and higher orders multipoint methods to solve (1). It is clear that the higher the order of the method is, the higher the rate of convergence and its operational cost will be. This requires finding an equilibrium between the high rate of convergence and the operational cost. In spite of this fact, these methods are important because many applications such as stiff systems of equations require quick convergence of their solution methods. Based on Ostrowski fourth order multipoint method, Sharma and Guha [5] described a one parameter family of sixth order methods for solving (1). Each member of the family requires three evaluations of the given function and one evaluation of its derivative per iteration. Starting with an initial approximation *x*_0_ near to the root *α*, a one parameter family of sixth order methods proposed in [10] is given for *k* = 0,1, 2,…, by

(4) $$\begin{array}{c} w_{k}=x_{k}-\frac{f\left(x_{k}\right)}{f^{\prime }\left(x_{k}\right)},\\ z_{k}=w_{k}-\frac{f\left(w_{k}\right)}{f^{\prime }\left(x_{k}\right)}\frac{f\left(x_{k}\right)+\beta f\left(w_{k}\right)}{f\left(x_{k}\right)+\left(\beta -2\right)f\left(w_{k}\right)},\\ x_{k+1}=z_{k}-\frac{f\left(z_{k}\right)}{f^{\prime }\left(x_{k}\right)}\frac{f\left(x_{k}\right)-f\left(w_{k}\right)+\gamma f\left(z_{k}\right)}{f\left(x_{k}\right)-3f\left(w_{k}\right)+\gamma f\left(z_{k}\right)}. \end{array}$$

Clearly, it requires three functions and one derivative evaluation per iteration. Starting with an initial approximation *x*_0_ near to the root *α*, Miquel and Díaz-Barrero [11] developed a variant of Ostrowski fourth order multipoint method defined for *k* = 0,1, 2,…, by

(5) $$\begin{array}{c} z_{k}=x_{k}-\frac{f\left(x_{k}\right)}{f^{\prime }\left(x_{k}\right)},\\ x_{k+1}=z_{k}-\frac{f\left(z_{k}\right)}{f^{\prime }\left(x_{k}\right)}\frac{f\left(x_{k}\right)}{f\left(x_{k}\right)-2f\left(z_{k}\right)},\\ {\tilde{x}}_{k+1}=x_{k+1}-\frac{f\left(x_{k+1}\right)}{f^{\prime }\left(x_{k}\right)}\frac{f\left(x_{k}\right)}{f\left(x_{k}\right)-2f\left(z_{k}\right)}. \end{array}$$

It is clear that this variant requires an additional evaluation of function *f* at the point iterated by Ostrowski's fourth order method; consequently, the local order of convergence is improved from four to six. Kou and Li [4] established a variant of Jarratt method for solving (1). Starting with a suitably chosen *x*_0_, it is given for *k* = 0,1, 2,…, by

(6) Jf(xk)=3f′(yk)+f′(xk)6f′(yk)−2f′(xk),zk=xk−Jf(xk)f(xk)f′(xk),xk+1=zk−((f(zk))     ×(32Jf(xk)f′(yk)      +(1−32Jf(xk))f′(xk))−1),

where *y*_*k*_ = *x*_*k*_ − (2/3)(*f*(*x*_*k*_)/*f*′(*x*_*k*_)). Per iteration the new method adds the evaluation of the function at another point in the procedure iterated by Jarratt method. As a consequence, the local order of convergence is improved from four for Jarratt method to six for the new method.

In this paper, a new iterative method is described for finding the real roots of nonlinear equations in *R*. Starting with a suitably chosen *x*_0_, the method generates a sequence of iterates converging to the root. The convergence analysis is provided to establish its sixth order of convergence. The number of iterations and the total number of function evaluations used to get a simple root are taken as performance measure of our method. The efficacy of the method is tested on a number of numerical examples and the results obtained are summarized in tables. It is observed that our method is superior to Newton's method and other sixth order methods described in [4, 10, 11].

The paper is organized as follows.  Section 1 is the introduction. In Section 2, the proposed method and its convergence analysis are described. The numerical examples are worked out in Section 3. Finally, conclusions are included in Section 4.

## 2. The Proposed Method and Its Convergence Analysis

In this section, we shall describe our sixth order method and its convergence analysis for finding a simple root *α* of (1). The following definition is used for convergence of our method.

Let *α* ∈ *R*, *x*_*k*_ ∈ *R*, *k* = 0,1, 2…. Then the sequence {*x*_*k*_} is said to converge to *α* if

(7) $$\begin{array}{c} \underset{n\to \infty }{\lim}\left|x_{k}-\alpha \right|=0. \end{array}$$

If, in addition, there exist a constant *c* ≥ 0, an integer *k*_0_ ≥ 0, and *p* ≥ 0 such that for all *k* > *k*_0_

(8) $$\begin{array}{c} \left|x_{k+1}-\alpha \right|\leq c{\left|x_{k}-\alpha \right|}^{p}, \end{array}$$

then {*x*_*k*_} is said to converge to root *α* with order at least *p*. If *p* = 2 or 3, the convergence is said to be quadratic or cubic, respectively.

When *e*_*k*_ = *x*_*k*_ − *α* is the error at the *k*th iterate, the relation

(9) $$\begin{array}{c} e_{k+1}=ce_{k}^{p}+O\left(e_{k}^{p+1}\right) \end{array}$$

is called the error equation. The value of *p* is called the order of this method.

Starting with a suitably chosen initial approximation *x*_0_, the fourth order multipoint method described in [2] for solving (1) is given for *k* = 0,1, 2,…, by

(10) $$\begin{array}{c} z_{k}=x_{k}-\frac{f\left(x_{k}\right)}{f^{\prime }\left(x_{k}\right)},\\ x_{k+1}=z_{k}-\frac{f\left(z_{k}\right)}{f^{\prime }\left(x_{k}\right)}\frac{f\left(x_{k}\right)}{f\left(x_{k}\right)-2f\left(z_{k}\right)}. \end{array}$$

Extending this method, our sixth order method is given for *k* = 0,1, 2,…, by

(11) $$\begin{array}{c} z_{k}=x_{k}-\frac{f\left(x_{k}\right)}{f^{\prime }\left(x_{k}\right)},\\ x_{k+1}=z_{k}-\frac{f\left(z_{k}\right)}{f^{\prime }\left(x_{k}\right)}\frac{f\left(x_{k}\right)}{f\left(x_{k}\right)-2f\left(z_{k}\right)},\\ {\tilde{x}}_{n+1}=x_{k+1}-\frac{f\left(x_{k+1}\right)\left(x_{k+1}-z_{k}\right)}{f\left(x_{k+1}\right)-f\left(z_{k}\right)}. \end{array}$$

Now, we shall establish the convergence analysis of our method.

**Theorem 1**

Let *f* : *R* → *R* be continuous derivatives up to third order in *R*. If *f*(*x*) has a simple root *α* in *R* and *x*_0_ is near to *α*, then the error in the method given by (11) satisfies

(12) $$\begin{array}{c} e_{k+1}=c_{2}^{2}\left(c_{2}^{3}-c_{2}c_{3}\right)e_{k}^{6}+O\left(e_{k}^{7}\right), \end{array}$$

where *e*_*k*_ = *x*_*k*_ − *α* and *c*_*j*_ = (*f* ^*j*^(*α*)/*j*!*f*′(*α*)), *j* = 2,3,….

**Proof**

Let *e*_*k*_ = *x*_*k*_ − *α* be the error in the iterate *x*_*k*_. Using Taylor's series expansion, we get

(13) f(xk)=f′(α)[ek+c2ek2+c3ek3     +c4ek4+c5ek5+O(ek6)],

(14) f′(xk)=f′(α)[1+2c2ek+3c3ek2     +4c4ek3+5c5ek4+O(ek5)].

Dividing (13) by (14), we get

(15) f(xk)f′(xk)=[ek−c2ek2+(2c22−2c3)ek3  +(−4c23+7c2c3−3c4)ek4+O(ek5)],

for

(16) zk=xk−f(xk)f′(xk),=α+c2ek2+(2c3−2c22)ek3 +(4c23−7c2c3+3c4)ek4+O(ek5).

Now expanding *f*(*z*_*k*_) about the *α*, we get

(17) f(zk)=f′(α)[(zk−α)+c2(zk−α)2     +c3(zk−α)3+c4(zk−α)4     +O(ek5)],=f′(α)[c2ek2+(2c3−2c22)ek3     +(5c23−7c2c3+3c4)ek4+O(ek5)],f(xk)−2f(zk)=ek−c2ek2+(4c22−3c3)ek3 +(−10c23+14c2c3−5c4)ek4+O(ek5).

Therefore,

(18) f(xk)f(xk)−2f(zk)=1+2c2ek+(−2c22+4c3)ek2 −2(2c2c3−3c4)ek3+O(ek4).

Also,

(19) f(zk)f′(xk)=c2ek2+(−4c22+2c3)en3 +(13c23−14c2c3+3c4)ek4+O(ek5).

Multiplication of (18) and (19) gives

(20) f(zk)f′(xk)f(xk)f(xk)−2f(zk)=c2ek2+(−2c22+2c3)ek3 +3(c23−2c2c3+c4)ek4+O(ek5).

(21) xk+1=zk−f(zk)f′(xk)f(xk)f(xk)−2f(zk),=α+(c23−c2c3)ek4;

that is,

(22) $$\begin{array}{c} x_{k+1}-\alpha =\left(c_{2}^{3}-c_{2}c_{3}\right)e_{k}^{4}+O\left(e_{k}^{5}\right). \end{array}$$

Now, the Taylors expansion of *f*(*x*_*k*+1_) about the *α* is given by

(23) f(xk+1)=f′(α)[(xk+1−α)+(xk+1−α)2+⋯],=f′(α)[(c23−c2c3)ek4+O(ek5)].

Therefore,

(24) f(xk+1)(xk+1−zk)f(xk+1)−f(zk)=(c23−c2c3)en4−2(2c24−4c22c3+c32+c2c4)ek5 +O(ek6).

By using (21)–(24) in third step of proposed method, we get the following error equation:

(25) $$\begin{array}{c} {\tilde{e}}_{k+1}=c_{2}^{3}\left(c_{2}^{2}-c_{3}\right)e_{k}^{6}+O\left(e_{k}^{7}\right). \end{array}$$

Thus, the sixth order of convergence of the method is established.

## 3. Numerical Examples

In this section, our proposed method (11) is tested on the following functions for different values of the initial approximations *x*_0_:

(26) f1(x)=x3−x2−1 α=1.465571231876768,f2(x)=x2−ex−3x+2 α=0.257530285439860,f3(x)=x10−1 α=1,f4(x)=x5+x−10000 α=6.308777129972688,  f5(x)=ex2+7x−30−1 α=3.

The comparison of the number of iterations (NN) taken by Newton's method (NM), Neta's method [10] (NEM), Kou and Li's method [4] (KLM), Miquel and Díaz-Barrero's method [11] (GBM), and our proposed method (PM) to find *α* correct up to 15 decimal places is given in Table 1. The word NC implies that the method is not convergent. The total number of function evaluations (NOFE) taken by these methods is compared with our proposed method in Table 2. A - indicates that no function evaluations are counted since the method does not converge.

From Tables 1 and 2, one can easily observe that the proposed method takes the less number of iterations and number of function evaluations compared with the other methods. For some functions, our method either is superior or behaves similarly to with other methods compared. In some cases, the proposed method converges whereas other methods diverge.

## 4. Conclusions

A new iterative method is described for finding the real roots of nonlinear equations in *R*. Starting with a suitably chosen *x*_0_, the method generates a sequence of iterates converging to the root. The convergence analysis is provided to establish its sixth order of convergence. The number of iterations and the total number of function evaluations used to get a simple root are taken as performance measure of our method. The efficacy of the method is tested on a number of numerical examples and the results obtained are summarized in tables. It is observed that our method is superior to Newton's method and other sixth order methods described in [4, 10, 11].

## Figures and Tables

**Table 1:** Comparison of number of iterations (NN).

Functions	*x*_0_	NN				
		NM	GBM	KLM	NEM	PM
*f*_1_	0.5	12	8	NC	12	8
*f*_2_	2	5	3	4	3	2
	3	6	2	3	3	2
*f*_3_	0.5	43	15	18	NC	14
	0.8	10	3	3	NC	3
*f*_4_	4	8	4	NC	9	3
	4.5	7	3	NC	3	3
*f*_5_	3.25	8	3	4	4	3
	3.5	12	5	5	6	4

**Table 2:** Comparison of number of function evaluations (NOFE).

Functions	*x*_0_	NOFE				
		NM	GBM	KLM	NEM	PM
*f*_1_	0.5	24	32	—	48	32
*f*_2_	2	10	12	16	12	8
	3	12	8	12	12	8
*f*_3_	0.5	86	60	72	—	56
	0.8	20	12	12	—	12
*f*_4_	4	16	16	—	36	12
	4.5	14	12	—	12	12
*f*_5_	3.25	16	12	16	16	12
	3.5	24	20	20	24	16
